# Diagnostic evaluation and short-term outcome as indicators of long-term prognosis in horses with findings suggestive of inflammatory bowel disease treated with corticosteroids and anthelmintics

**DOI:** 10.1186/1751-0147-56-35

**Published:** 2014-06-03

**Authors:** Ritva Kaikkonen, Kati Niinistö, Benjamin Sykes, Marjukka Anttila, Satu Sankari, Marja Raekallio

**Affiliations:** 1 Department of Equine and Small Animal Medicine, Faculty of Veterinary Medicine, University of Helsinki, P.O. Box 57, FI-00014 Helsinki, Finland; 2 Faculty of Veterinary Medicine, Estonian University of Life Sciences, Tartu, Estonia; 3 Pathology Unit, EVIRA, Mustialankatu 3, 00790 Helsinki, Finland; 4 Current work address: Animagi Equine Clinic Oulu, Äimärautiontie 5, 90400 Oulu, Finland

**Keywords:** Horse, Weight loss, Colic, IBD, Total protein, Albumin, D-xylose

## Abstract

**Background:**

Recurrent colic and unexplained weight loss despite good appetite and adequate feeding and management practices are common conditions in the horse. However, little information has been published on the systematic diagnostic evaluation, response to treatment, prognostic factors or outcome of either presentation. The aims of this study were to 1) identify possible prognostic indicators and 2) report the short- and long-term response to treatment with corticosteroid therapy of a variety of horses with a presumptive diagnosis of inflammatory bowel disease (IBD).

Thirty-six horses with a history of recurrent colic and/or unexplained weight loss were screened with a detailed clinical, clinicopathological and diagnostic imaging examination. Twenty horses were subsequently selected that had findings consistent with inflammatory bowel disease based on the fulfilment of one or more of the following additional inclusion criteria: hypoproteinaemia, hypoalbuminaemia, malabsorption, an increased intestinal wall thickness on ultrasonographic examination or histopathological changes in rectal biopsy. These 20 horses were treated with a standardized larvicidal anthelmintic regime and a minimum of three weeks of corticosteroid therapy.

**Results:**

The initial response to treatment was good in 75% (15/20) of horses, with a 3-year survival rate of 65% (13/20). The overall 3-year survival in horses that responded to initial treatment (12/15) was significantly higher (*P* = 0.031) than in those that did not respond to initial treatment (1/5). The peak xylose concentration was significantly (*P* = 0.048) higher in survivors (1.36 ± 0.44 mmol/L) than non-survivors (0.94 ± 0.36 mmol/L).

**Conclusions:**

The overall prognosis for long-term survival in horses with a presumptive diagnosis of IBD appears to be fair to moderate, and the initial response to anthelmintic and corticosteroid therapy could be a useful prognostic indicator. The findings of the present study suggest that a low peak xylose concentration in absorption testing is associated with a less favourable prognosis, supporting the use of this test.

## Background

Recurrent colic and weight loss are conditions in the horse that can present either in combination or as separate problems. Numerous reasons exist for both presentations, including management and feeding practices, intestinal diseases such as parasitism and neoplasias [1,2] and various non-intestinal diseases [1]. Inflammatory bowel disease (IBD) is another potential cause of both recurrent colic and weight loss that has been histologically classified into the different subtypes of lymphocytic-plasmacytic enterocolitis (LPE), granulomatous enteritis (GE), multisystemic eosinophilic epitheliotropic disease (MEED), diffuse eosinophilic enteritis (DEE) and idiopathic focal eosinophilic enteritis (IFEE) [3-6]. IBD has previously been reported to cause a variety of clinical presentations, from malabsorption and weight loss to diarrhoea and colic. In particular, eosinophilic enteritis has been reported to cause colic, while weight loss, hypoproteinaemia, hypoalbuminaemia and carbohydrate malabsorption have all been associated with LPE, MEED and GE [4,5].

Currently, diagnostic evaluation of equine gastrointestinal disease includes clinical and dental examination, routine haematology and biochemistry. Where these fail to identify the pathology, abdominal ultrasonography, carbohydrate absorption tests and histopathology of rectal mucosal biopsies may be useful [1]. The use of rectal mucosal biopsies is appealing, as they are minimally invasive and easy to obtain with no specialized equipment, and histopathology has been demonstrated to be potentially useful in determining the underlying disease process [7,8]. Alternatively, gastric and duodenal mucosal biopsies can be taken via endoscopy, or full thickness intestinal biopsies can be taken via exploratory celiotomy. However, these require specialized equipment that is not always available or affordable, which limits their usefulness.

Indirect tests of intestinal function, such as D-xylose and glucose absorption, have been described in the horse. These tests can provide useful information, particularly on small intestinal function. The D-xylose absorption test is considered more accurate than the glucose absorption test, because D-xylose is not normally present in the horse and the serum concentration of D-xylose is not affected by factors such as metabolic status, stress or excitement, in contrast to glucose [1,9-13].

The retrospective evaluation of horses with weight loss has recently been reported by Metcalfe *et al.*[14], but at present there is a paucity of systematic prospective studies evaluating the results, and identification of risk factors, from routine examination (history and clinical evaluation), measurement of carbohydrate absorption, abdominal ultrasonography and histopathological findings from rectal biopsy in horses with recurrent colic or weight loss. Furthermore, limited information exists regarding the treatment response of various enteropathies to corticosteroid therapy, although the prognosis of IBD is generally regarded as guarded to poor [4,8,15-17]. Finally, the long-term prognosis for horses with a presumptive diagnosis of IBD that respond to initial treatment with corticosteroid therapy has not yet been reported.

The aims of the study were to 1) identify prognostic indicators and 2) report the short- and long-term response of a variety of horses with signs suggestive of IBD to treatment with corticosteroid and anthelmintic therapy.

## Methods

### Horses

The prospective cohort clinical study was performed between 1 May 2004 and 31 December 2005 at the Equine Hospital of the Faculty of Veterinary Medicine, University of Helsinki, and Hyvinkää Equine Hospital, Finland. Long-term survival was reported for a 3-year period following the discontinuation of treatment. Horses with a history of recurrent colic, as defined by at least two episodes of colic requiring veterinary attention during the preceding 12 months [18], or anamnestic information regarding unexplained weight loss or an inability to gain weight despite adequate feeding and management practices, were considered eligible for initial screening for inclusion in the study. Furthermore, to be eligible for screening, horses were required to be ≥2 years of age and have received adequate feeding, deworming and dental care as determined by the examining veterinarian. Horses that presented with acute colic or that had previously undergone colic surgery were excluded, as were horses receiving corticosteroid therapy at the time of assessment or in the six months preceding assessment. The study was approved by the Ethical Committee of the University of Helsinki (UH number 89–04). Informed consent from the owner, or the trainer acting as an agent for the owner, was obtained at the time of enrolment in the study.

### Diagnostic evaluation

A thorough history was obtained, including signalment and management. The initial examination included a basic clinical examination, determination of body weight with scales, lameness examination, oral examination, palpation per rectum, a complete blood count, fibrinogen measurement, serum biochemistry (globulins were counted by subtracting serum albumin from total protein), thoracic and abdominal radiography, transabdominal ultrasonography, peritoneal fluid analysis, urinalysis, a faecal flotation test for parasites, a D-xylose absorption test [19], gastroscopy and duodenoscopy [20], as well as a rectal mucosal biopsy and histopathology, as described by Lindberg *et al.*[7]. The body condition score was determined by two clinicians, as described by Henneke *et al.*[21], and ranged from 1 to 9, where 1 was extremely emaciated and 9 extremely overweight.

In addition to having fulfilled the criteria for initial screening to be included in the final study, horses had to fulfil at least one of the following criteria: hypoproteinaemia (defined as a serum total protein concentration of less than 54 g/L), hypoalbuminaemia (defined as a serum albumin concentration of less than 28 g/L), a peak plasma xylose concentration of less than 1.33 mmol/L [1], increased intestinal wall thickness (>4 mm) according to ultrasonographic assessment [22] or changes in the rectal mucosal biopsy suggestive of IBD. Horses were excluded from the final study if another, non-IBD cause of the presenting clinical signs was identified. This included horses with significant intestinal sand accumulation [23], severe gastric ulceration (grades 3–4/4), evidence of infectious disease as indicated by changes in haematology and/or fibrinogen, and serum biochemical changes consistent with renal or hepatic failure.

### Therapeutic protocols

All horses included in the study were initially treated with the same protocol. Anthelmintics were started with five consecutive days of fenbendazole (Axilur, MSD Animal Health) 10 mg/kg *per os* (PO) once daily, followed by a single dose of ivermectin at 0.2 mg/kg and praziquantel at 1 mg/kg (Equimax, Virbac de Portugal) PO on day 6. Prednisolone (Prednisolon 40 mg, Leiras) at 1 mg/kg PO once daily was started on day 1 and continued until the first follow-up examination was performed, after approximately three weeks of treatment.

On follow-up, the horses were clinically evaluated, body weight was determined by scales, and the tests previously identified as abnormal were repeated. Further medication was decided at the treating clinician’s discretion according to the findings from the follow-up examination and based on the following guidelines: if the original complaint(s) (weight loss and/or recurrent colic) had clinically improved and the results from any/all previously abnormal tests (D-xylose absorption, histopathology of rectal mucosal biopsy, intestinal wall thickness as determined by ultrasonography, hypoalbuminaemia and/or hypoglobulinaemia) had improved or normalised, corticosteroid therapy was gradually tapered and then discontinued. Horses that had shown minimal or no improvement were changed to dexamethasone (Dexametason 1.5 mg, Orion Pharma) at 0.1 mg/kg PO once daily with subsequent treatment at the treating clinician’s discretion. Horse owners were interviewed by telephone about their horse’s condition and the possible reoccurrence of any symptoms approximately 3 and 6 months following the discontinuation of treatment. The national horse register database was used to trace horses for long-term survival at three years after the discontinuation of treatment.

### Statistical analysis

The Shapiro-Wilk test was used to evaluate whether the data were normally distributed. Pearson correlations were calculated between the normally distributed parameters, and the two-tailed Student’s t-test was used for comparisons between horses that were or were not alive three years after treatment. Spearman's correlation and the Mann–Whitney U-test were used for nonparametric data. A two-tailed Fischer’s exact test was used to compare the response to initial treatment between survivors and non-survivors. Significance was set at *P* < 0.05. Post-hoc power calculations were performed using online data management software [24].

## Results

### Horses

Thirty-six client-owned horses met the criteria for initial screening, with 20 horses subsequently included in the final study. Five of the 20 horses included in the final study had a primary complaint of recurrent colic, nine horses were included due to weight loss and six presented with a history of both recurrent colic and weight loss.

### Diagnostic evaluation

Histopathology of the rectal mucosal biopsy was considered abnormal in 5/13 and 3/7 of survivors and non-survivors, respectively. Intestinal thickening (>4 mm), as determined by ultrasound, was present in 6/13 and 4/7 of survivors and non-survivors, respectively. These proportions did not significantly differ between survivors and non-survivors. The proportion of horses with a peak D-xylose concentration below the normal threshold of 1.33 mmol/L did not differ between survivors (8/13) and non-survivors (6/7). However, the mean peak D-xylose concentration was significantly higher in survivors (1.36 ± 0.44) than non-survivors (0.94 ± 0.36) (*P* = 0.048). Faecal flotation was negative in all horses. The presumptive diagnosis of the IBD subtype was based on the history, clinical presentation and the results of the diagnostic work-up, with particular emphasis placed on histopathological changes in the rectal mucosal biopsy.

### Treatment and follow up

Treatment was attempted in all horses and all returned for re-evaluation at approximately three weeks. Five horses failed to demonstrate satisfactory improvement in their clinical or clinicopathological parameters in the first weeks and were changed to dexamethasone therapy as outlined above. The remaining 15 horses were considered to have improved and corticosteroid therapy was tapered and discontinued.

Seven of the 20 horses died during the three-year follow-up period, with death related to the original problem of weight loss or recurrent colic in all but one horse (Table 1). Of these seven horses, three had initially responded well to treatment, while four had responded poorly. The overall 3-year survival rate in horses that responded to initial treatment (12/15: 80%) was significantly higher (*P* = 0.031) than in horses that did not respond to initial treatment and were subsequently changed to dexamethasone (1/5: 20%). Five of the non-surviving horses underwent post-mortem examination, and in four of these horses this examination confirmed the presumptive diagnosis of inflammatory bowel disease. Table 2 presents the age, body composition score, serum protein, albumin and globulin concentration, and peak D-xylose concentrations at enrolment, as well as the length of treatment for survivors and non-survivors. The peak D-xylose concentration correlated significantly with serum total protein (r = 0.45, *P* < 0.001) and globulin concentrations (r = 0.61, *P* = 0.005), but not with serum albumin concentration (r = −0.19, *P* = 0.41). The power of the study to detect a difference between survivors and non-survivors was 49.1%, 12.1% and 62.6% for serum total protein, serum albumin and serum globulin concentrations, respectively.

**Table 1:** Presumptive IBD subtype (based on the clinical picture and findings from the diagnostic work-up) and post-mortem diagnoses (when available) of non-survivors (n = 7), and length of survival after the discontinuation of treatment

**Presumptive diagnosis**	**Time when died/euthanized**	**Indication**	**Post-mortem diagnosis**
Lymphocytic-plasmacytic enteritis	During the treatment (day 26)	Progressive weight loss, animal welfare	Lymphocytic-plasmacytic enteritis
Eosinophilic enterocolitis	2 days after discontinuing the treatment	Colic	Idiopathic small intestinal muscular hypertrophy
Eosinophilic enterocolitis	22 days after discontinuing the treatment	Death due to acute cardiac failure	Eosinophilic enterocolitis Acute cardiac failure
Eosinophilic enterocolitis	30 days after discontinuing the treatment	Colic	Not performed
Lymphocytic-plasmacytic enteritis	120 days after discontinuing the treatment	Progressive weight loss	Lymphocytic-plasmacytic enteritis
Eosinophilic enterocolitis	7 months 13 days after discontinuing the treatment	Recurrent colic	Not performed
Eosinophilic enterocolitis	1 year 8 months after discontinuing the treatment	Recurrent colic	Eosinophilic enterocolitis

**Table 2:** Means ± SD (min–max) of the age, body composition score, serum protein, albumin and globulin concentrations, and peak xylose concentration, the numbers of horses with each abnormal finding and the length of treatment in long-term (3 years) survivors and non-surviving horses

	**Survivors (n = 13)**	**Non-survivors (n = 7)**	***P***
Age (years)	9.7 ± 4.5 (3–17)	10.0 ± 5.3 (4–20)	0.893
Body score	3.9 ± 1.4 (2–7)^1^	3.6 ± 0.5 (3–4)	0.598
S-protein (g/L)	61.6 ± 8.3 (44.2–77.2)	53.4 ± 11.9 (30.6–69.6)	0.086
S-albumin (g/L)	33.1 ± 5.5 (20.3–40.8)	31.4 ± 8.7 (14.7–39.4)	0.843
S-globulin (g/L)	28.5 ± 7.5 (21.3–46.4)	22.0 ± 6.8 (15.5–30.9)	0.073
Peak xylose (mmol/L)	1.36 ± 0.44 (0.85–2.27)	0.94 ± 0.36 (0.27–1.37)	0.048
Length of treatment (d)	51.1 ± 13.2 (22–69)	71.1 ± 32.8 (43–120)^2^	0.065

^1^Data missing from two surviving horses.

^2^One horse is not included in this calculation as it was euthanized on humane grounds after 26 days of treatment.

## Discussion

In the present study, the response to anthelmintic and corticosteroid therapy over a three-week period was demonstrated to be a useful predictor of the outcome in horses with a presumptive diagnosis of IBD. The long-term prognosis for horses in the present study was fair to moderate, with 65% of the horses surviving at least three years. This is in contrast to the current literature, in which the response of IBD to corticosteroid treatment is generally considered to be poor [4,8,16,17]. Possible reasons for this discrepancy, including case selection, case definition and the role of anthelmintic therapy in the outcome, warrant discussion.

Anthelmintic therapy was performed regardless of the negative results of faecal analysis, as the faecal flotation test underestimates the parasite burden when there are more larval stages than adults, and is unable to detect encysted cyathostomes [25]. Furthermore, it has been postulated that endoparasites may have a role in triggering the disease process of inflammatory bowel disease [26-28]. As such, the treatment of endoparasitism in suspected IBD cases is logical. Fenbendazole was chosen as the therapeutic agent, as it is efficacious against encysted cyathostomes when used as described above [29], and there was no known resistance towards fenbendazole in the geographical area in which the study was performed.

Although both hospitals involved in the study were referral centres, the case load at both facilities is a broad representation of the clinical spectrum, with a large proportion of the case load effectively comprising first opinion cases. The inclusion of first opinion cases may at least partly explain the better outcome observed in the study than previously reported. Similarly, most of the cases in the present study that were presumptively diagnosed with IBD were less severely affected than the confirmed cases described in the literature [8,30,31]. This may have biased the population studied towards animals that were more likely to respond to treatment than those previously reported from true referral populations [4,8,16,17]. Alternatively, it is possible that the response to treatment seen in some cases was due to the larvicidal anthelmintic treatment that was included in the protocol rather than the corticosteroids. All horses in the present study had a history of adequate anthelmintic treatment, and no significant parasite burdens were detected on faecal analysis, reducing the likelihood that endoparasites were a major factor. However, neither factor excludes parasitism as a potential diagnosis, especially cyathostomiasis, as discussed above.

The authors recognise that the definition of IBD used in this study is broad and may inadvertently encompass other disease conditions such as low-grade cyathostomiasis. However, despite the limitations in ruling out parasitism and achieving a definitive diagnosis, we believe that present study accurately reflects the clinical presentation of weight loss and recurrent colic seen in practice. As such, we argue that the results of the study are applicable to the clinical setting. The finding that the response to initial treatment is predictive of long-term survival is of particular importance and direct clinical relevance. The development of clearer guidelines for the diagnosis of IBD would be beneficial in defining inclusion criteria for future prospective studies.

The results of this study support the use of the D-xylose test in the diagnostic evaluation of recurrent colic and weight loss, with inadequate D-xylose absorption the only significant clinicopathological prognosticator of survival identified. Importantly, the horse’s diet can influence the D-xylose curve, with horses fed a high-energy diet reported to have lower peak plasma concentrations [32]. Additionally, prolonged fasting (72–96 h) can lower the peak concentration of D-xylose [33] and warrants consideration in the interpretation of the test results. In the present study, all the horses were fed in a similar manner, with none receiving a high-energy diet, and no horses were fasted for a prolonged period before testing. As such, these factors are unlikely to have influenced the accuracy of the test.

In the present study, peak D-xylose concentrations were not associated with the serum albumin concentration, but were associated with the serum globulin concentration. This contradicts the existing literature, wherein it is commonly assumed that horses with malabsorption will also have a low serum albumin concentration, since the rate of albumin production is slower compared to globulin production [34]. This assumption is supported by a recent study in which both hypoproteinaemia and hypoalbuminaemia, but not hypogammaglobulinaemia, were associated with non-survival in horses with unexplained weight loss [14]. The small number of horses in the present study is a significant limitation, and the power of the study to detect a difference between survivors was only 49.1%, 12.1% and 62.6% for serum total protein, serum albumin and serum globulin concentrations, respectively. As such, the risk of a type II error was relatively high in the present study. The trend towards a difference between survivors and non-survivors in serum total protein, which is consistent with a recent report [14], and in serum globulins, which has not previously been reported, warrants consideration in the interpretation of results, although care should be taken in drawing firm conclusions.

Interestingly, despite hypoalbuminaemia previously being identified as a significant risk factor for non-survival in a previous study [14], the data from the present study do not support a similar conclusion. The reasons for this discrepancy are not immediately apparent. Both the present study and the previous study by Metcalfe *et al.*[14] reporting the outcome of chronic gastrointestinal disease were limited by small numbers. The risk of both type I and type II statistical errors increases as a function of decreasing sample size, and consequentially power [35]. As such, the inability to find an association between serum albumin and the outcome in the present study, or the association previously reported by Metcalfe *et al.*[14], could be erroneous due to a type II or type I statistical error, respectively. Considering this, a larger prospective clinical study is warranted to further investigate the relationship between clinicopathological parameters and the outcome before any firm conclusions can be drawn. However, combining the results of the present study with the previous observations of Metcalfe *et al.*[14], serum total protein appears to be the most consistent clinicopathological predictor of the outcome.

Histopathological changes in the rectal biopsy were detected in less than half of the cases studied, but the findings of the present study suggest that when present, changes in rectal mucosal histopathology were in line with subsequent post-mortem findings. This supports their use in the diagnostic evaluation of weight loss and/or recurrent colic cases.

## Conclusions

The overall prognosis for long-term survival in horses with a presumptive diagnosis of IBD appears to be fair to moderate. The initial response to anthelmintic and corticosteroid therapy could be a useful prognostic indicator, and the findings of the present study suggest that a low peak xylose concentration in absorption testing is associated with a less favourable prognosis, supporting the use of this test.

## Competing interests

The authors declare that they have no competing interests.

## Authors’ contributions

RK participated in study design, data collection and manuscript preparation. KN collected the data and revised the manuscript. BS participated in study design, data collection and manuscript revision. MA performed the histopathology and participated in manuscript revision. SS participated in study design, laboratory analysis and manuscript revision. MR participated in study design, laboratory analysis and manuscript revision. All authors read and approved the final version of the manuscript.
